# Obstacle Avoidance and Target Acquisition for Robot Navigation Using a Mixed Signal Analog/Digital Neuromorphic Processing System

**DOI:** 10.3389/fnbot.2017.00028

**Published:** 2017-07-11

**Authors:** Moritz B. Milde, Hermann Blum, Alexander Dietmüller, Dora Sumislawska, Jörg Conradt, Giacomo Indiveri, Yulia Sandamirskaya

**Affiliations:** ^1^Institute of Neuroinformatics, University of Zurich and ETH Zurich Zurich, Switzerland; ^2^Neuroscientific System Theory, Department of Electrical and Computer Engineering, Technical University of Munich Munich, Germany

**Keywords:** neuromorphic controller, obstacle avoidance, target acquisition, neurorobotics, dynamic vision sensor, dynamic neural fields

## Abstract

Neuromorphic hardware emulates dynamics of biological neural networks in electronic circuits offering an alternative to the von Neumann computing architecture that is low-power, inherently parallel, and event-driven. This hardware allows to implement neural-network based robotic controllers in an energy-efficient way with low latency, but requires solving the problem of device variability, characteristic for analog electronic circuits. In this work, we interfaced a mixed-signal analog-digital neuromorphic processor ROLLS to a neuromorphic dynamic vision sensor (DVS) mounted on a robotic vehicle and developed an autonomous neuromorphic agent that is able to perform neurally inspired obstacle-avoidance and target acquisition. We developed a neural network architecture that can cope with device variability and verified its robustness in different environmental situations, e.g., moving obstacles, moving target, clutter, and poor light conditions. We demonstrate how this network, combined with the properties of the DVS, allows the robot to avoid obstacles using a simple biologically-inspired dynamics. We also show how a Dynamic Neural Field for target acquisition can be implemented in spiking neuromorphic hardware. This work demonstrates an implementation of working obstacle avoidance and target acquisition using mixed signal analog/digital neuromorphic hardware.

## 1. Introduction

Collision avoidance is one of the most basic tasks in mobile robotics that ensures safety of the robotic platform, as well as the objects and users around it. Biological neural processing systems, including relatively small ones such as those of insects, are impressive in their ability to avoid obstacles robustly at high speeds in complex dynamical environments. Relatively simple neuronal architectures have already been proposed to implement robust obstacle avoidance (e.g., Blanchard et al., 2000; Iida, 2001; Rind and Santer, 2004), while probably the most simple conceptual formulation of a neuronal controller for obstacle avoidance is the famous Braitenberg vehicle (Braitenberg, 1986). When such *neuronal* control architectures are implemented on a conventional computer, analog sensor signals are converted and stored in digital variables. A large number of numerical computations are performed then, which are required to model the involved neuronal dynamics in software.

Neuromorphic hardware offers a physical computational substrate for directly emulating such neuronal architectures in real time (Indiveri et al., 2009; Furber et al., 2012; Benjamin et al., 2014; Chicca et al., 2014), enabling low latency and massively parallel, event-based computation. Neuromorphic electronic circuits can implement dynamics of neurons and synapses using digital (Furber et al., 2012) or analog (Benjamin et al., 2014; Qiao et al., 2015) designs and allow for arbitrary connectivity between artificial neurons. The analog implementations of artificial neural networks are particularly promising, due to their potential smaller size and lower power consumption figures than digital systems (for a review see Indiveri et al., 2011; Hasler and Marr, 2013). But these features come at a price of precision and reliability. Indeed, with analog designs, the device mismatch effects (i.e., variation in properties of artificial neurons across the device) have to be taken into account for the development of robust functional architectures (Neftci et al., 2011).

A promising strategy for taking these issues into account is to implement the mechanisms used in *biological neural networks*, which face the same problem of using an unreliable computing substrate that consists of noisy neurons and synapses driven by stochastic biological and diffusion processes. These biological mechanisms include adaptation and learning, but also using *population coding* (Ermentrout, 1998; Pouget et al., 2000; Averbeck et al., 2006) and *recurrent connections* (Wilson and Cowan, 1973; Douglas et al., 1995) to stabilize behaviorally relevant decisions and states against neuronal and sensory noise. In this work, we show that by using the population-coding strategy in a mixed signal analog/digital neuromorphic hardware, it is possible to cope with the variability of its analog circuits and to produce reliably the desired behavior on a robot.

We present a first proof of concept implementation of such a neuromorphic approach to robot navigation. Specifically, we demonstrate a *reactive vision-based* obstacle avoidance strategy using a neurally-inspired event-based Dynamic Vision Sensor (DVS) (Lichtsteiner et al., 2006) and a Reconfigurable On-Line Learning (ROLLS) neuromorphic processor (Qiao et al., 2015). The proposed architecture is event-driven and uses the neural populations on the ROLLS device to determine the steering direction and speed of the robot based on the events produced by the DVS. In the development phase, we use a miniature computer Parallella^1^ solely to manage the traffic of events (spikes) between the neuromorphic devices, and to store and visualize data from the experiments. The Parallella board can be removed from the behavioral loop in target applications, leading to a purely neuromorphic implementation. In this paper, we demonstrate the robustness and limits of our system in a number of experiments with the small robotic vehicle “Pushbot^2^” in a robotic arena, as well as in an unstructured office environment.

Several neuromorphic controllers for robots were developed in the recent years, e.g., a SpiNNacker system (Furber et al., 2012) was used to learn sensory-motor associations with robots (Conradt et al., 2015; Stewart et al., 2016), a neural-array integrated circuit was used to plan routes in a known environment (Koziol et al., 2014), three populations of analog low-power subthreshold VLSI integrate-and-fire neurons were employed to control a robotic arm (Perez-Peña et al., 2013). Our system goes along similar lines and realizes a reactive robot navigation controller that uses a mixed signal analog/digital approach, and exploits the features of the ROLLS neuromorphic processor.

In this work we follow a dynamical systems—attractor dynamics—approach to robot navigation (Bicho et al., 2000), which formalizes one of the famous Braitenberg vehicles (Braitenberg, 1986). The neuronal architecture in our work is realized using a number of neuronal populations on the neuromorphic device ROLLS. The dynamical properties of neuronal populations and their interconnectivity allow to process a large amount of sensory signals in parallel, detecting the most salient signals and stabilizing these detection decisions in order to generate robustly closed-loop behavior in real-world unstructured and noisy environments (Sandamirskaya, 2013; Indiveri and Liu, 2015). Here, we demonstrate the feasibility of deployment of a neuromorphic processor for the closed loop reactive control. We found several limitations of the simple Braitenberg-vehicle approach and suggest extensions of the simple architecture that solve these problems, leading to robust obstacle avoidance and target acquisition in our robotic setup.

## 2. Materials and methods

**Figure 1:**
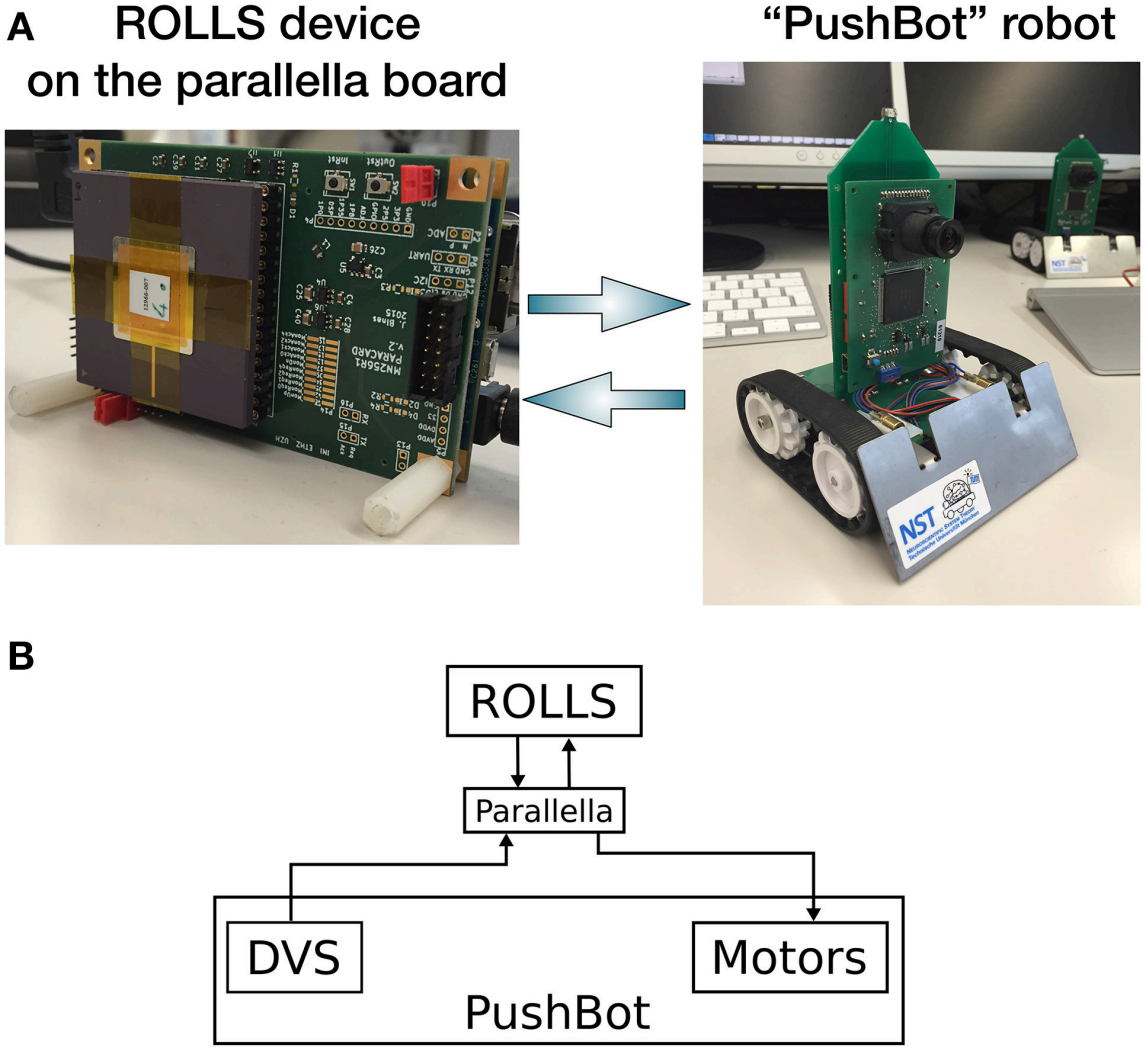
**(A)** Information flow between the three main components: ROLLS, Parallella, and the Pushbot, in particular, its sensor DVS and two motors. **(B)** The hardware setup used in this work: the neuromorphic processor ROLLS is interfaced to a miniature computer Parallella, which communicates with the Pushbot robot over a dedicated WiFi network.

The experimental setup used in this work consists of the Pushbot robotic vehicle with an embedded DVS camera (eDVS) and the ROLLS neuromorphic processor. A miniature computing board Parallella is used to direct the flow of events between the robot and the ROLLS. Figure 1A shows the components of our hardware setup, while Figure 1B shows the information flow between different hardware components.

The Pushbot communicates with the Parallella board via a wireless interface for receiving motor commands and for sending address-events produced by the DVS. Using a dedicated WiFi network, we achieve communication latency below 10 ms, which was enough to demonstrate functionality of our system at speeds, possible with the Pushbot.

The ROLLS device is interfaced to the Parallella board using an embedded FPGA, which is used to configure the neural network connectivity on the chip and to direct stimulating events to neurons and synapses in real time. The Parallella board runs a simple program that manages the stream of events between the neuromorphic processor and the robot.

### 2.1. The ROLLS neuromorphic processor

The ROLLS is a mixed signal analog/digital neuromorphic chip (Qiao et al., 2015) that comprises 256 spiking silicon neurons, implemented using analog electronic circuits which can express biologically plausible neural dynamics. The neurons can be configured to be fully connected with three sets of synaptic connections: an array of 256 × 256 non-plastic (“programmable”) synapses, 256 plastic (“learnable”) synapses that realize a variant of the Spike-Timing-Dependent Plasticity (STDP) rule (Mitra et al., 2009), and 4 additional (“virtual”) synapses that can be used to receive external inputs. In this work, only the programmable synapses were used for setting up the neuronal control architecture, as no online-learning was employed for the navigation task.

**Figure 2:**
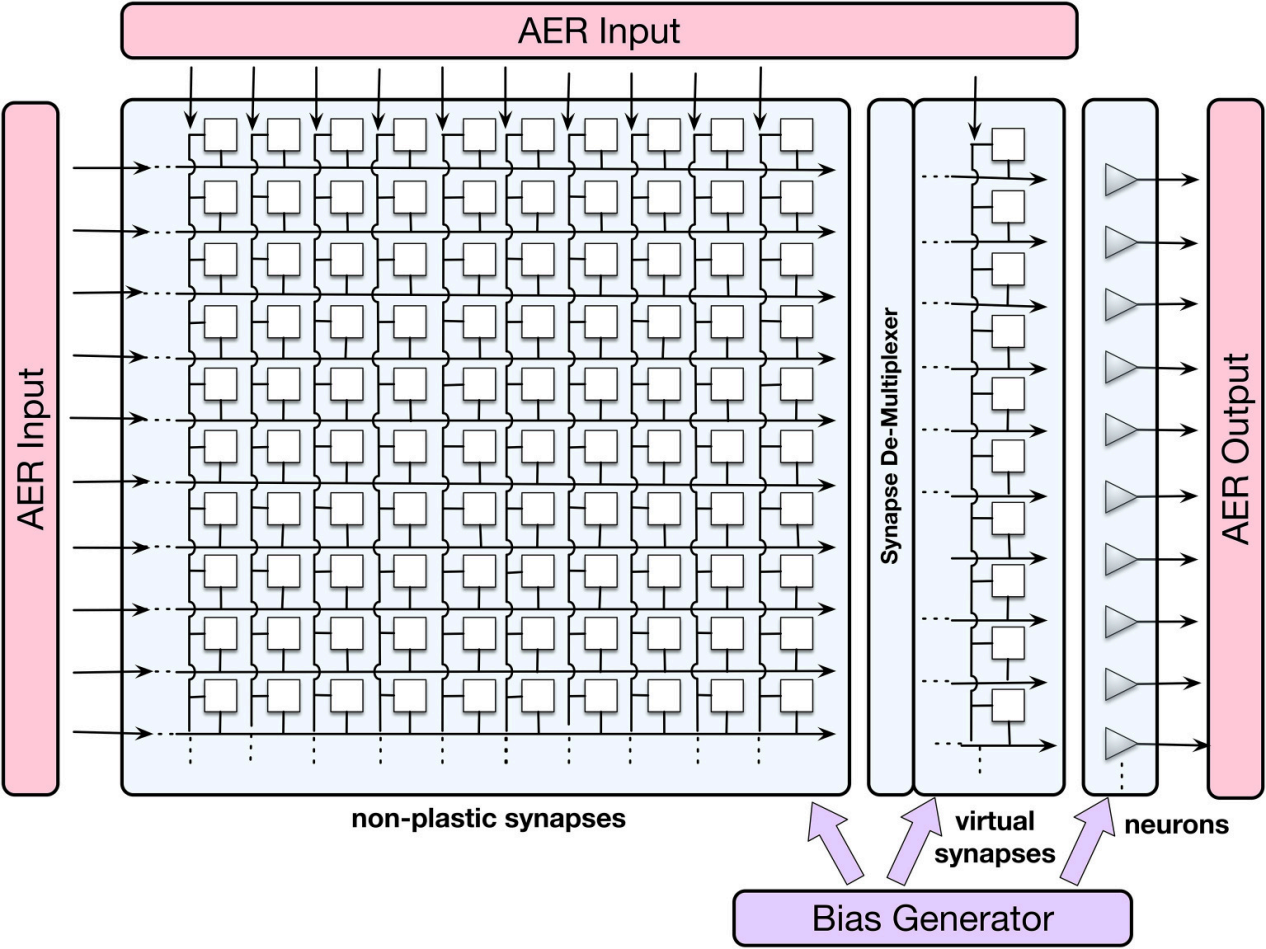
The schematic visualization of neurons (grey triangles), non-plastic and virtual synapses (white squares), as well as input-output interfaces and bias generator of the ROLLS chip. Each neuron on the chip (presynaptic neuron) sends its output spikes to 256 non-plastic synapses, which, if set active, can route these spikes to any of the neurons on the chip (postsynaptic neurons). The connectivity matrix allows for all-to-all connectivity, but also other configurations. AER is a digital Address-Event Representation, used to communicate spikes (it consists of an index of the spike-emitting neuron).

Figure 2 shows a block diagram of the ROLLS device, in which 256 spiking neurons, implemented using analog electronic circuits (Indiveri et al., 2006), are shown as triangles on the right, and 256 × 256 non-plastic (“programmable”) synapses, which can be used to create a neuronal architecture on the ROLLS, as well as 256 “virtual” synapses used to stimulate neurons externally, are shown as white squares. A digital Address Event Representation (AER) circuitry allows to stimulate neurons and synapses on the chip, as well as to read-out spike events off chip; a temperature-compensated digital bias-generator allows to control parameters of analog electronic neurons and synapses, such as the refractory period or membrane time constant.

The programmable synapses share a set of biases that determine their weight values, their activation threshold, and time constants. These three parameters determine the synaptic strength and dynamics of the respective connection between two neurons. A structural limitation of the hardware is that each synapse can only assume one of eight possible weight values (four excitatory and four inhibitory values). This means that in a neuronal architecture, several different populations might have to share weights, which limits the complexity of the architecture. ROLLS consumes ~4 mW of power in typical experiments, run here. The ROLLS parameters (biases) used in this work are listed in the Appendix (Supplementary Material, Appendix A).

### 2.2. The DVS camera

**Figure 3:**
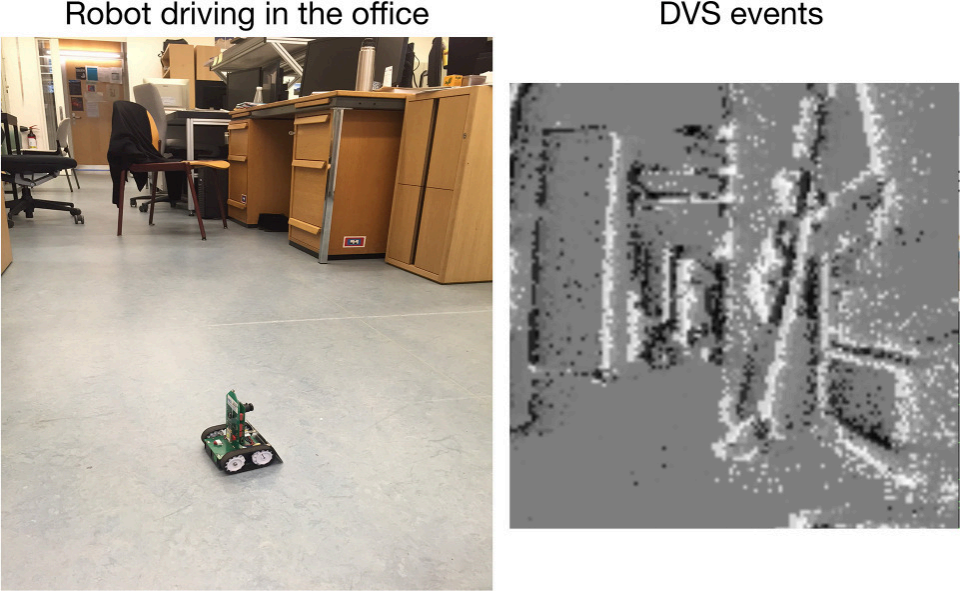
The Pushbot robot driving in the office **(left)** and a visualization of the DVS output **(right)**, accumulated over 0.5 s.

The Dynamic Vision Sensor (DVS) is an event-based camera, inspired by the mammalian retina (Lichtsteiner et al., 2006; Liu and Delbruck, 2010). Figure 3 shows a typical output of the DVS camera accumulated over 0.5 s (right) from the Pushbot robot driving in the office (left).

Each pixel of the DVS is sensitive to a relative temporal contrast change. If such change is detected, each pixel sends out an *event* at the time in which the change was detected (asynchronous real-time operation). Each event *e* is a vector: *e* = (*x, y, ts, p*), where *x* and *y* define the pixel location in retinal reference frame, *ts* is the time stamp, and *p* is the polarity of the event. The event polarity encodes whether the luminance of the pixel increased (an “on” event) or decreased (an “off” event). All pixels share a common transmission bus, which uses the Address Event Representation (AER) protocol to transmit the address-events off chip.

The AER representation and asynchronous nature of communication makes this sensor low power, low latency, and low-bandwidth, as the amount of data transmitted is very small (typically, a very small subset of pixels produce events). Indeed, if there is no change in the visual scene, no information is transmitted off the camera. If a change is detected, it is communicated instantaneously, taking only a few microseconds to transfer the data off-chip.

For the obstacle avoidance scenario, important properties of the DVS are its low data rate, high dynamic range, and small sensitivity to lighting conditions (Lichtsteiner et al., 2006). The challenges are noise, inherent in the sensor, its inability to detect homogeneous surfaces, and relatively small spatial resolution (128 × 128 pixels), as well as a limited field of view (60°). New versions of DVS are currently available, which would further improve performance of the system. Moreover, more sophisticated object-detection algorithms for DVS are currently being developed (Moeys et al., 2016).

The embedded version of the DVS (eDVS) camera (Müller and Conradt, 2011) used in this work uses an ARM Cortex microcontroller to initializes the DVS, capture events, send them to the wireless network, and to receive and process commands for motor control of the Pushbot.

### 2.3. Neuromorphic robot

The robot used in this work is the mobile autonomous platform Pushbot, which consists of a 10 × 10 cm chassis with two motors driving two independent tracks for propulsion (left and right). The predominant component on the small robot is an eDVS (Section 2.2), which acquires and provides sensory information and controls actuator output, including the robot's motors, through its embedded microcontroller. The sensor's integrated 9 DOF IMU reports changes of velocity and orientation. The robot actuators include a buzzer, two parallel, horizontal forward laser pointers and an LED on top, which all can show arbitrary activation patterns. The Pushbot is powered by 4 AA-batteries, which ensure ~2 h operation time.

The robot communicates through WLAN at up to 12 Mbps, which allows remote reading of sensory data (including events from the eDVS) and setting velocities with a latency <10 ms. The Pushbot robot is too small to carry the current experimental hardware setup. In principle, however, it is possible to place the ROLLS chip directly on a robot, removing the WiFi latency.

### 2.4. Spiking neural network architecture

**Figure 4:**
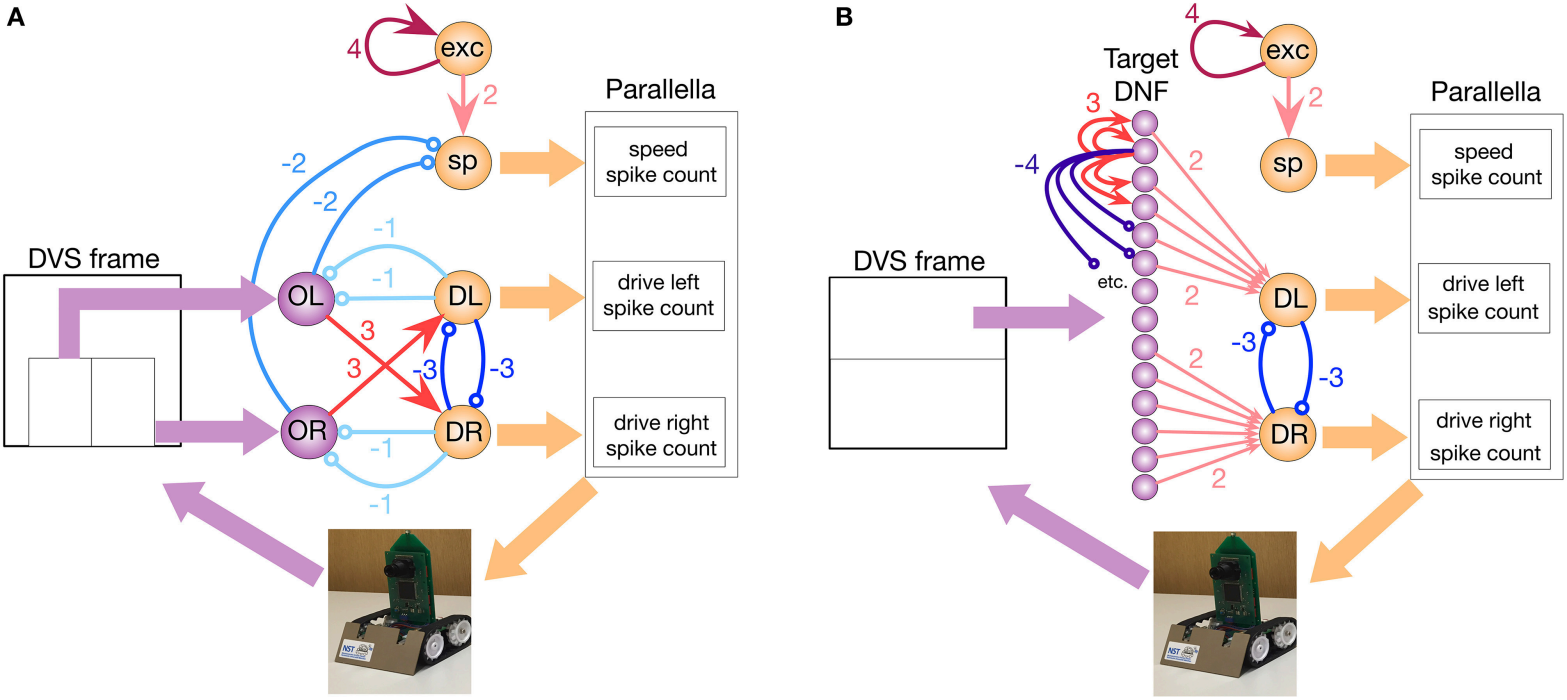
The implemented neuronal architectures for obstacle avoidance and target acquisition. **(A)** Obstacle avoidance: Violet OL and OR circles represent obstacle detecting neuronal populations. Orange DL and DR circles are motor driving populations. sp is the speed-setting population, and exp is the constantly firing population that sets the default speed. Thin arrows show excitatory non-plastic connections realized on the ROLLS chip, whereas colors and numbers show the weights (the exact value of the weight is set by the biases, listed in Table 2) in Appendix B (Supplementary Material). **(B)** Target acquisition: Same notations. The violet line of circles shows a DNF population that represent targets. On the chip, both architectures are realized at the same time.

The core of the system presented here is a simple neural network architecture that is realized in the ROLLS device and allows the robot to avoid obstacles and approach a simple target. The “connectionist” scheme of the obstacle avoidance part of the architecture is shown in Figure 4A, while the scheme of the target acquisition architecture is shown in Figure 4B.

For obstacle avoidance, we configured two neuronal populations of 16 neurons each to represent a sensed obstacle to the right (“obstacle right,” or OR) and to the left (“obstacle left,” or OL) from the robot's heading direction. Each neuron in the OL and OR populations receives a spike for each DVS pixel that produces an event in the left (right) part of the sensor, respectively (we used the lower half of the sensor for obstacle avoidance). The spiking neurons in the two obstacle populations sum up the camera events according to their neuronal integrate-and-fire dynamics (equations can be found in Appendix B (Supplementary Material)). If enough events arrive from the same neighborhood, the respective neuron will fire, otherwise it will ignore events that are caused by the sensor noise. Thus, the obstacle representing neuronal populations achieve basic filtering of the DVS events. The output spikes of the neuronal populations signal the detection of an object in the respective half of the field of view.

Each of the obstacle detecting neuronal populations is connected to a motor population “drive left, DL” or “drive right, DR” (with 16 neurons per population). Consequently, if an obstacle is detected on the right, the drive left population is stimulated, and vice versa. The drive populations inhibit each other, implementing a winner-take-all dynamics. Thus, a decision about the direction of an obstacle-avoiding movement is taken and stabilized at this stage by the dynamics of neuronal populations on the chip.

The *drive* populations, in their turn, inhibit both obstacle detecting populations, since during a turning movement of the robot, many more events are generated by the DVS, compared to those generated during translational motion. This inhibition compensates for this expected increase in the input rate, similar to the motor re-afferent signals in biological neural systems (Dean et al., 2009). This modification of the simple Braitenberg vehicle principle is required to enable robust and fast behavior.

The speed of the robot is controlled by a neuronal population, “speed, sp,” which receives input from a constantly firing “exc,” excitatory population. The latter group of neurons has strong recurrent connections and continually fires when triggered by a transient activity pulse. In an obstacle-free environment, the speed population sets a constant speed for the robot. The obstacle detecting populations OL and OR inhibit the speed population, making the robot slow down if obstacles are present. The decreasing speed ensures a collision-free avoidance maneuver.

These six populations comprise only 96 neurons, and represent all that is needed to implement the obstacle avoidance dynamics in this architecture (Figure 4A).

The control signals sent to the robot are, first, the angular velocity, *v*_*a*_, that is proportional to the difference in the number of spikes per neuron emitted between the two drive populations (Equation 1), and, second, the forward velocity, calculated based on the number of spikes per neuron emitted by the speed population (Equation 2):

(1) $$\begin{array}{r} v_{a}=c_{turn}\left(\frac{N_{DL}^{spike}}{N_{DL}^{n}}-\frac{N_{DR}^{spike}}{N_{DR}^{n}}\right), \end{array}$$

(2) $$\begin{array}{r} v_{f}=c_{speed}\frac{N_{sp}^{spike}}{N_{sp}^{n}}, \end{array}$$

where $N_{XX}^{spike}$ are the numbers of spikes, obtained from the respective populations [drive left (DL), drive right (DR), and speed (sp)] in a fixed time-window, we used 500 and 50 ms in an improved version); $N_{XX}^{n}$ is the number of neurons in the respective population; and *c*_*turn*_ and *c*_*speed*_ are turn- and speed-factors (user-defined constants), respectively.

Thus, we used neural population dynamics to represent angular and translational velocities of the robot and used the firing rate of the respective populations of neurons as the control variable.

#### 2.4.1. Dynamic neural field for target representation

To represent targets of the navigation dynamics, we use a Dynamic Neural Fields (DNFs) architecture as defined in Bicho et al. (2000). DNFs are population-based models of dynamics of large homogeneous neuronal populations, which have been successfully used in modeling elementary cognitive function in humans (Schöner and Spencer, 2015), as well as in implementing cognitive representations for robots (Erlhagen and Bicho, 2006; Bicho et al., 2011; Sandamirskaya et al., 2013). DNFs can be easily realized in neuromorphic hardware by setting a winner-take-all (WTA) connectivity network in a neural population (Sandamirskaya, 2013). Each neuron in a soft WTA network has a positive recurrent connection to itself and to its 2–4 nearest neighbors, implementing the lateral excitation of the DNF interaction kernel. Furthermore, all neurons have inhibitory connections to the rest of the WTA network, implementing the global inhibition of a DNF. These inhibitory connections can be either direct, as used here, or be relayed through an inhibitory population, which is a more biologically plausible structure.

In our architecture, we select 128 neurons on the ROLLS chip to represent visually perceived targets. Each neuron in this population receives events from the upper half of each column of the 128 × 128 sensor frame from the eDVS and integrates these events according to its neuronal dynamics: only events that consistently are emitted from the same column lead to firing of the neuron. The nearby neurons support each other's activation, while inhibiting further neurons in the WTA population (Figure 4B).

This connectivity stabilizes localized blobs of most salient sensory events, filtering out sensor noise and objects that are too large (inhibition starts to play role within object representation) or too small (not enough lateral excitation is engaged). Thus, the WTA connectivity stabilizes the target representation. The target in our experiments was a blinking LED of the second robot, which was detected in the DNF realized on the ROLLS. While this target could be easily detected since the blinking LED produces many events, more sophisticated vision algorithms are being developed to pursue an arbitrary target (Moeys et al., 2016).

The target population was divided in three regions: neurons of the DNF that receive inputs to the left from midline of the DVS frame drive the “drive left” population, whereas neurons that receive input from the right half of the DVS frame drive the “drive right” population. We did not connect the central 16 neurons of the target DNF to the drive populations to ensure more smooth target pursue when the target is in the center of the DVS frame (Figure 4B).

#### 2.4.2. Combining obstacle avoidance and target acquisition

The two neuronal populations that ultimately determine the robot's steering direction (DR and DL) sum-up contributions from the obstacle-representing populations and the target-representing WTA population (Figure 4). The obstacle contribution is made effectively stronger than the target contribution by setting the ROLLS biases accordingly. Thus, in the presence of an obstacle in the robot's field of view, an obstacle avoidance maneuver is preferred.

**Figure 5:**
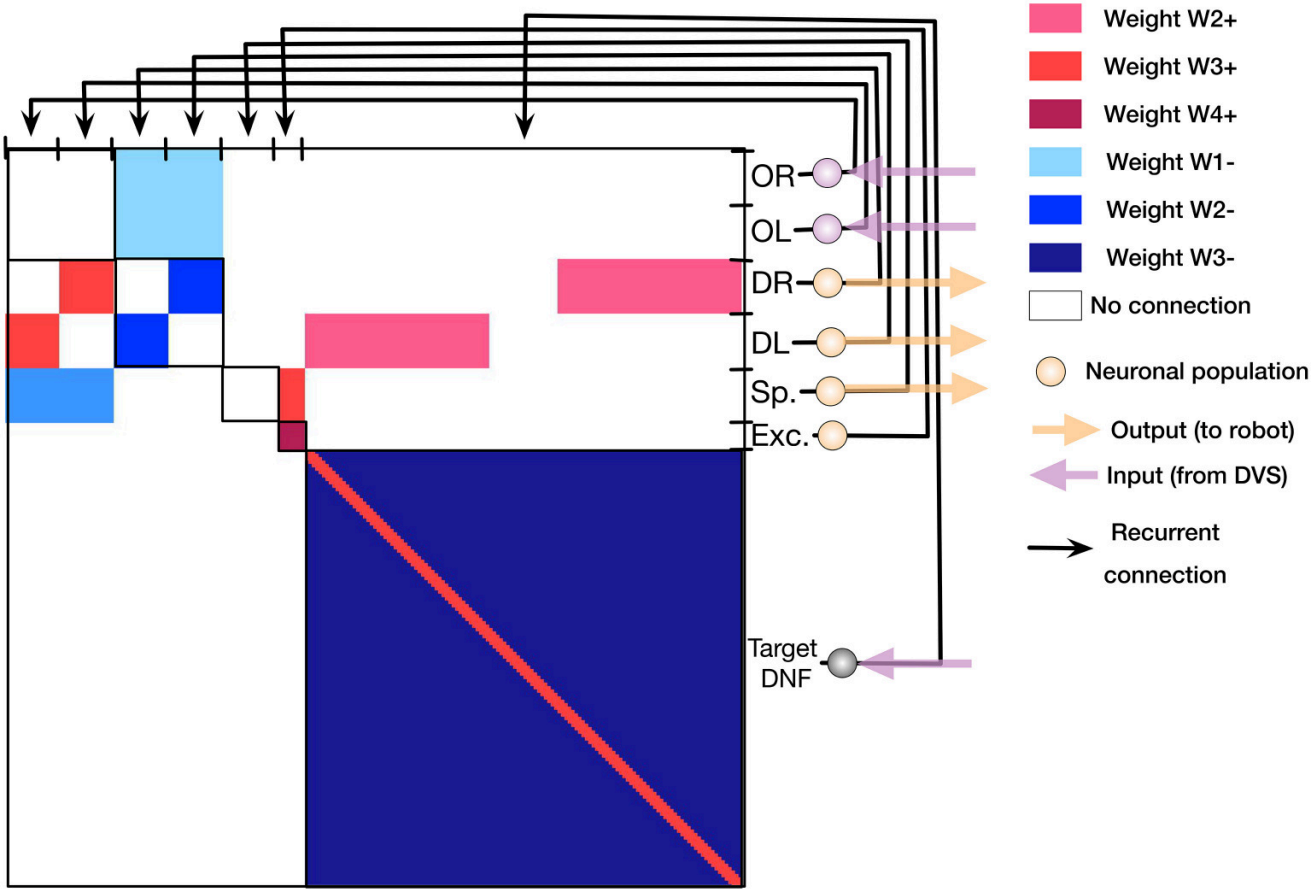
The synaptic connectivity matrix, configured on the ROLLS chip to implement the obstacle avoidance and target acquisition architectures. Colors encode different synaptic weights (red for positive and blue for negative connection weights) of the recurrent connections on the chip.

Figure 5 shows the connectivity matrix used to configure the non-plastic connections on the ROLLS chip to realize both obstacle avoidance and target acquisition. This plot shows the weights of non-plastic synapses on the ROLLS chip (blue being the negative weights and red the positive weights; the same color code is used for the different weights as in Figure 4), which connect groups of neurons (different populations, labeled on the right side of the figure) among each other. Within-group connections are marked with black squared frames on the diagonal of the connectivity matrix. Violet and orange arrows show inputs and outputs of the architecture, respectively.

This connectivity matrix is sent to the ROLLS device to configure the neuronal architecture on the chip, i.e., to “program” the device.

## 3. Demonstrations

We verified the performance of our system in a number of demonstrations, reported next. Overall, over 100 runs were performed with different parameter settings. In the following, we will provide an overview for the experiments and describe a few of them in greater detail to provide intuition of how the neural architecture works. For most experiments, we let the robot drive in a robotic arena with a white background and salient obstacles. We used a tape with a contrastive texture to make the walls of the arena visible to the robot. In four runs, we let the robot drive for several minutes freely in the office.

### 3.1. Probing the obstacle avoidance: a single static obstacle

**Figure 6:**
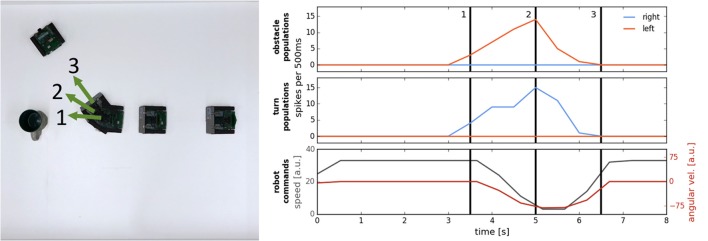
An example of an obstacle avoidance maneuver. **Left:** Overlay of video frames showing the trajectory of the robot. **Right:** activity of the neuronal populations on the chip (Top: the left and right obstacle detecting populations; Middle: the left and right drive populations), and the motor commands, sent to the robot (Bottom plot).

In the first set of experiments, we let the robot drive straight toward a single object (a colored block 2.5 cm wide and 10 cm high) and measured the distance from the object at which the robot crossed a virtual line perpendicular to the robot's initial heading direction, on which the object is located (e.g., see the distance between the robot and the “cup” object at the last position of the robot in Figure 6). We varied the speed factor of the architecture from 0.1 (~0.07 m/s) to 3.0 (~1 m/s) and have verified the effectiveness of the obstacle avoidance maneuver. Furthermore, we have increased the turning factor from 0.5 to 1.0 to improve performance at high speeds and have tested color-dependence of the obstacle perception with the DVS. Table 1 shows results of these measurements. Each trial was repeated 3 times and mean over the trials was calculated.

**Table 1:** Collision avoidance at different speeds: distance to the obstacle when crossing the obstacle-line (mean over 3 trials ± standard deviation in [cm]) at different speed- and turn-factors and for different colors of the obstacle.

**Speed/turn**	**0.1/0.5**	**0.5/0.5**	**1/0.5**	**1/1**	**1.5/1**	**2/1**	**3/1**
Blue	7.0 ± 1.0	10.3 ± 0.6	7.7 ± 1.5	19.3 ± 2.1	16.3 ± 3.3	10.8 ± 2.6	0^*^
Red	0^*^	2.3 ± 0.6	4.7 ± 0.6	10.7 ± 1.2	9.7 ± 3.5	5.0 ± 1.0	0^*^
Yellow	0^*^	0^*^	0^*^	7.0^*^ ± 6.1	0^*^	0^*^	0^*^

* *signifies trials when a collision happened*.

The table allows to note the following characteristics of the architecture at the chosen parametrization. First, the performance drops at very low speeds (speed factor 0.1), especially for red and yellow objects, due to an insufficient number of DVS events to drive the neuronal populations on ROLLS. Second, there is a trade-off between this effect and the expected decay in performance (in terms of the decreasing distance to the obstacle) with increasing speed. Thus, at a turning factor 0.5, best performance is achieved for the blue object at speed factor 0.5 and for the red object at speed factor 1. Distance to the obstacle can be further increased by increasing the turn factor. Thus, at turn factor 1 and speed factor 1 best performance (i.e., largest distance to the obstacle) can be achieved for both the blue and red objects. Yellow object provides too little contrast to be reliably perceived by the DVS in our set-up.

Figure 6 demonstrates how the neuronal architecture on the ROLLS chip realizes obstacle avoidance with the Pushbot. On the left, an overlay of video frames (recording the top view of the arena) shows the robot's trajectory when avoiding a single obstacle (here, a cup) in one of the runs. Numbers (1–3) mark important moments in time during the turning movement. On the right, summed activity of the neuronal populations on the ROLLS device is shown over time. The same moments in time are marked with numbers as in the left figure. In this case, already the obstacle detecting populations had a clear “winner”—the left population forms an increasing activity bump over time, which drives the “drive right” population, inducing a right turn of the robot. The bottom plot shows the commands that are sent to the robot (speed and angular velocity): the robot slows down in front of the obstacle and turns to the right.

**Figure 7:**
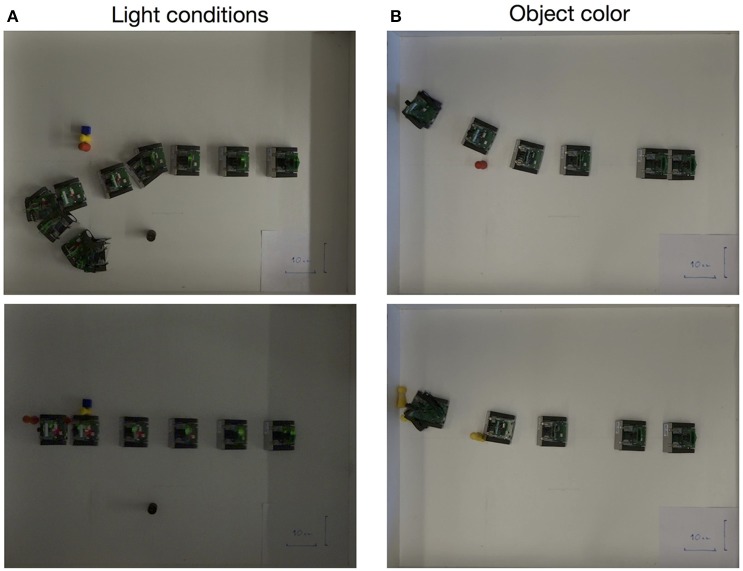
Exemplary experiments showing successful **(Top row)** and unsuccessful **(Bottom row)** obstacle avoidance maneuvers in different light conditions **(A)** and with obstacles of different colors **(B)**.

We have performed several further trials, varying the lighting conditions (normal, dark, very dark) and parameters of the architecture. Since the architecture uses the difference in spiking activity, induced by sensory events from the two halves of the visual space, avoiding a single obstacle works robustly, although the camera might miss objects with a low contrast (e.g., yellow block in our white arena). More advanced noise filtering would improve performance. While more extended version of the performed tests will be reported elsewhere, Figure 7 show results of some of the successful and unsuccessful runs.

### 3.2. Avoiding a pair of obstacles

We repeated the controlled obstacle avoidance experiment with two and three blocks in different positions. Each configuration was tested twenty times without crashes at speed 0.35 m/s (speed factor 0.5).

**Figure 8:**
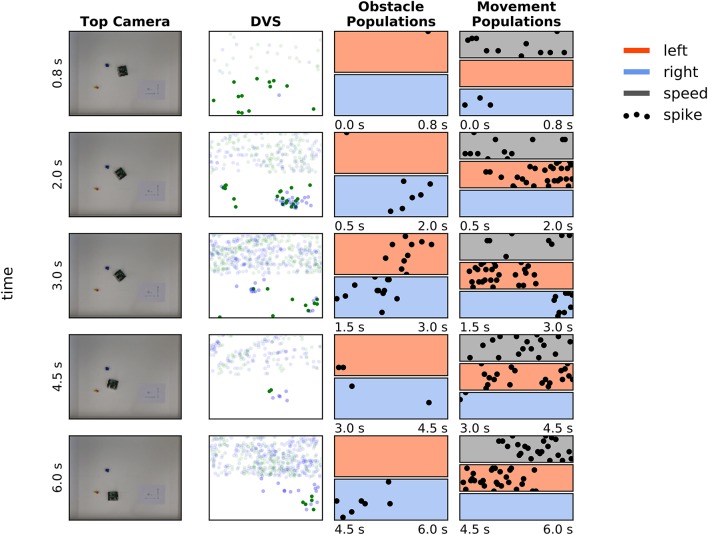
Avoiding a pair of obstacles. **First column:** Snapshots of four moments in time during avoidance of a cup, moved into the robot's trajectory. **Second column:** DVS “frames”—events, accumulated over a 0.5 s time window. Green dots are off events, blue dots are on events. Events in the upper part of the frame were not considered for the obstacle avoidance. **Third column:** Activity of the obstacle representing populations in 0.6–1.5 s before the camera snapshot in the first column was taken (red—left population (*n*_*OL*_), blue – right population (*n*_*O*_*R*); each population has 16 neurons). **Forth column:** Activity of the drive left (red), drive right (blue), and speed population on the ROLLS chip in the same time as on the plots in column 3.

Figure 8 shows an exemplary run that explains *how* the robot avoids a pair of obstacles. This example is important, since in the attractor dynamics approach to navigation, distance between the two objects determines a decision to move around or between the objects.

Snapshots from the overhead camera are shown on the left of Figure 8. Output of the DVS, accumulated in 500 ms time windows around the time when the snapshots were taken^3^, is shown in the second column, and the spiking activity of neuronal populations recorded from the ROLLS chip is shown in the two right-most columns. Activity is shown of the obstacle representing left (red) and right (blue) neuronal populations (third column), the left (red) and right (blue) drive populations, and the speed population (gray, forth column). Each of these populations has 16 neurons, dots represent their spikes^4^.

At the moment, depicted in the top row of Figure 8, the robot senses an obstacle on the right, although the DVS output is rather weak. Note that the neuronal population filters out sensory noise of the DVS and only detects events that cluster in time and in space. The robot turns left, driven by the activated *drive left* population and now the obstacle on the right becomes visible, providing a strong signal to the *right obstacle* population and, consequently, to the *drive left* population (second and third row). Eventually, the obstacle on the right dominates and the robot drives past both obstacles on the left side (forth row).

Thus, with the chosen parametrization of the neuronal network architecture, the robot tends to go around a pair of objects, avoiding the space between them. This behavior could be changed, making the connections between the obstacle representing populations and drive populations stronger. However, for a robot equipped with a DVS, such strategy is safer, since for homogeneous objects, the DVS can only sense the edges, where a temporal contrast change can be induced by the robot's motion. The robot thus might miss the central part of an object and avoiding pairs of close objects is a safer strategy. Adaptive connectivity that depends on the robot speed is also feasible.

### 3.3. Avoiding a moving obstacle

In these experiment, the robot is driving straight in the arena while we move an obstacle (a coffee mug) into its path. We repeat this experiment six times with varying speed factors (0.1–2) of the robot. The robot was capable to avoid collisions in all tested cases. In fact, avoiding a moving obstacle is more robust than avoiding a static obstacle because the moving obstacle produces more DVS events than a static one at the same robot speed.

**Figure 9:**
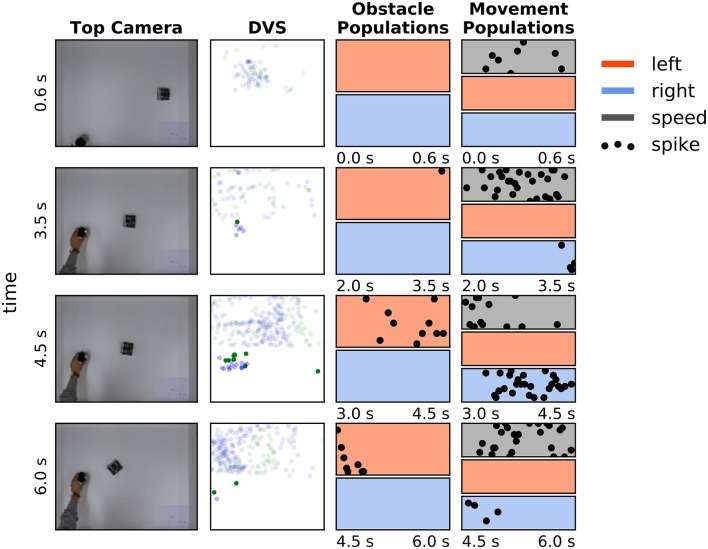
Avoiding a moving obstacle. The same arrangement is used as in Figure 8. See main text for the discussion.

Figure 9 shows how the robot avoids a moving obstacle. The same arrangement of plots was used as in Figure 8, described in Section 3.2. The robot was moving with *c*_*speed*_ = 0.5 (0.35 m/s) here, the cup was moved at ~0.20 m/s.

### 3.4. Cluttered environment

In the following set of experiments, we randomly placed obstacles (8–12 wooden pieces) in the arena and let the robot drive around at an average speed (0.35 m/s). We analyzed the performance of the architecture, suggesting a number of modifications to cope with its limitations.

**Figure 10:**
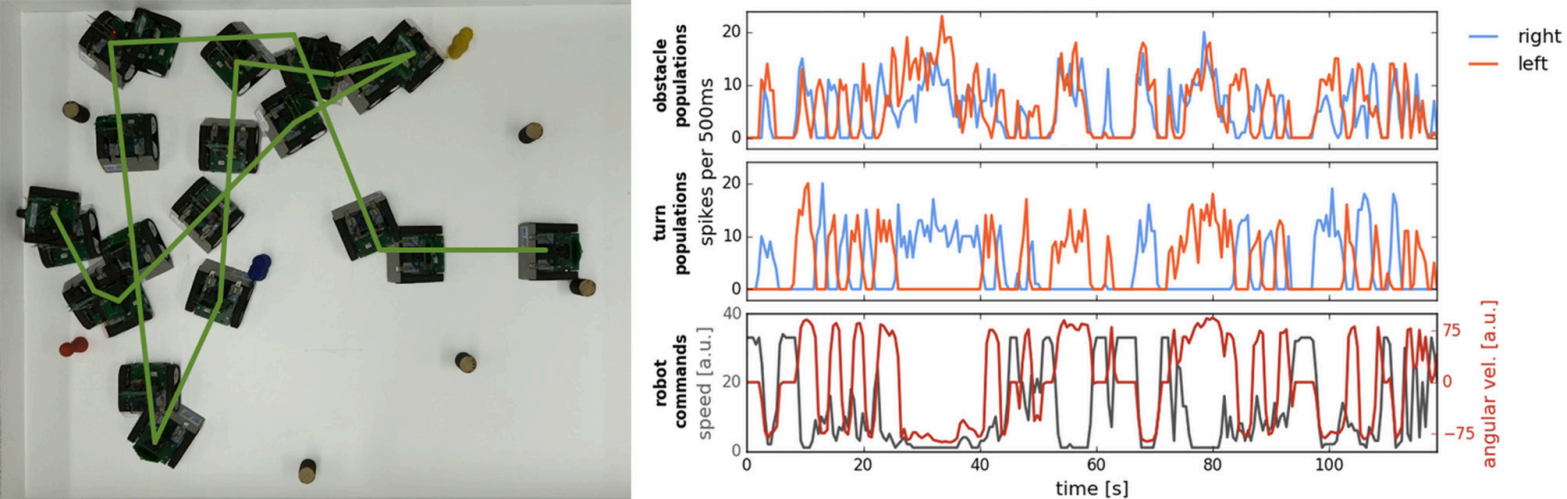
Navigation in a cluttered arena. **Left:** Overlayed frames from the video, recoding the robotic arena from the top. Green line markes the path of the robot. **Right:** Summed activation of neurons in populations on the ROLLS chip over the time of the experiment. Obstacle and turn (**left** and **right**) population are shown, as well as the commands sent to the robot (angular velocity and speed).

Figure 10 demonstrates behavior of the obstacle avoidance system in a cluttered environment. In particular, we let the robot drive in an arena, in which 8 obstacles were randomly distributed. The robot successfully avoids obstacles in its way with two exceptions: the robot touches the blue obstacle in the center of the arena, which entered the field of view too late for a maneuver, and also collides with the yellow object, which did not provide enough contrast to produce the required number of DVS events. These collisions point to two limitations of the current setup, which, first, uses single camera with a narrow field of view and, second, drops 80% of events to improve signal to noise ratio (the latter deprives performance for objects with low contrast against the background). Using more sophisticated noise filter would improve visibility of the faint obstacles. Note that we used rather small objects on these trials (blocks of 2 × 5 cm), which posed a challenge for the event-based detection, especially taking into account our very simplistic noise-reduction strategy.

**Figure 11:**
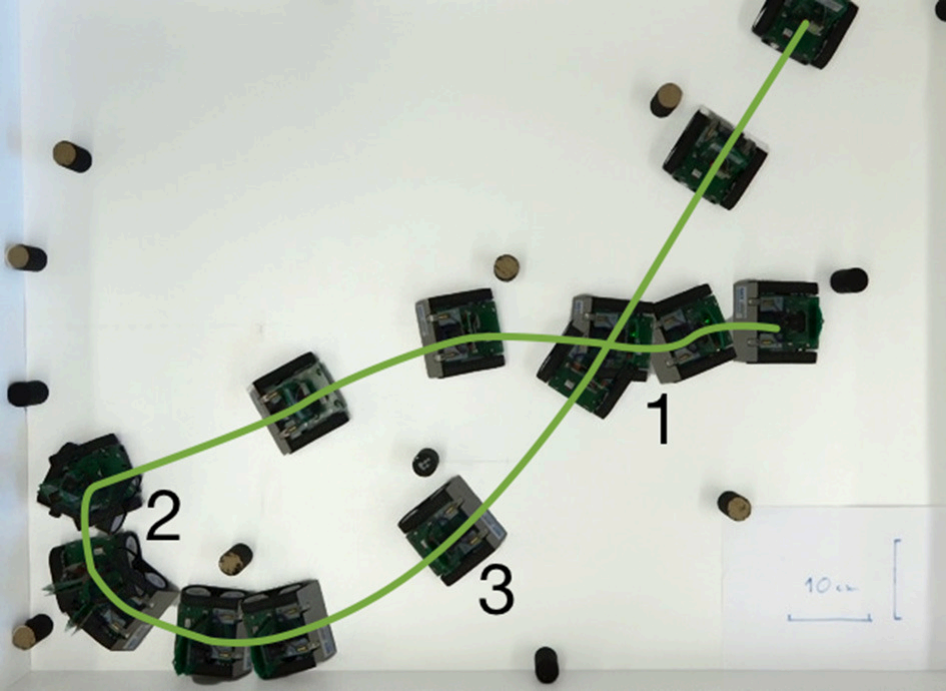
Successful run in a cluttered environment with a modified neuronal architecture. Overlay of the overhead-camera frames.

To improve behavior in a cluttered environment, we modified the architecture, adding two more populations on the ROLLS chip, which receive input from the inertia measurement unit of the Pushbot and which suppress obstacle populations when the robot is turning. Moreover, we replaced the homogeneous connections between the obstacle and the drive populations with graded connections that become stronger for obstacles detected in the center than in the periphery of DVS field of view. This allows the robot to make shorter avoidance maneuvers and avoid obstacles in a denser configuration at a higher speed. Figure 11 shows a successful run with the modified architecture. Here, we also changed the sampling mechanism used to calculate the robot commands, replacing a fixed time window with a running average. This allowed us to avoid obstacles in the cluttered environment without collisions at speed as high as 0.5 m/s.

### 3.5. Variability of behavior

Since behavior of our robot is controlled by activity of neuronal populations, implemented in analog neuromorphic hardware, the behavior of the robot has some variability, even when exactly the same parameters of the architecture and the same hardware biases are used. Despite this variability, the robot's goal—avoiding obstacles—remains fulfilled. Such variability of behavior can be used as a drive for exploration, which may be exploited in learning scenarios in more complex architectures, built on top of our elementary obstacle avoidance system.

**Figure 12:**
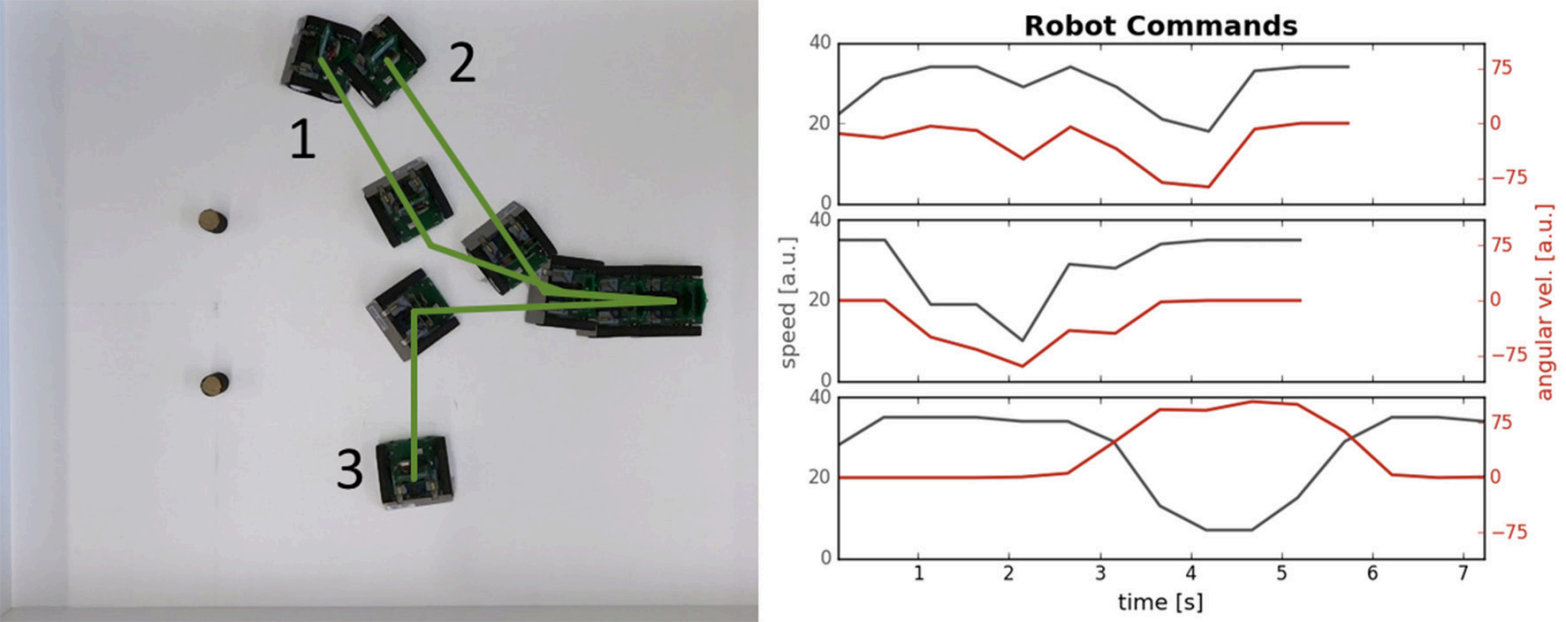
Variability of the robot's behavior. **Left:** Overlay of video camera frames recording the robot, avoiding a pair of obstacles; top view. Three different trials are recorded and overlayed here (trajectories are shown with green lines 1–3). **Right:** Velocity commands, received by the robot from the neuronal architecture (angular velocity and speed) for the three trials (from top to bottom).

Figure 12 demonstrates variability behavior of our neuronal controller. In the figure, we show three trials, in which the robot avoids a two-blocks configuration, starting from exactly the same position and with the same configuration of the neuronal controller (speed factor 0.5, turn factor 0.5). Mismatch in the neuronal populations implemented in analog neuromorphic hardware, variability of the DVS output, and its dependence on the robot's movements lead to strong differences in trajectories. In particular, in the case shown in Figure 12, the trajectories may bifurcate and the robot might avoid the two obstacles on the right, or on the left side.

### 3.6. Obstacle-avoidance in a real-world environment

Finally, we tried our architecture outside of the arena as well. The robot was placed on the floor in the office and drove around avoiding both furniture and people. The high amount of background activity compared to the arena did not diminish the effectiveness of the architecture: in four 0.5–1.5-min long trials, the robot only crashes once after it maneuvered itself into a dark corner under a table where the DVS sensor could not provide sufficient information to recognize obstacles.

**Figure 13:**
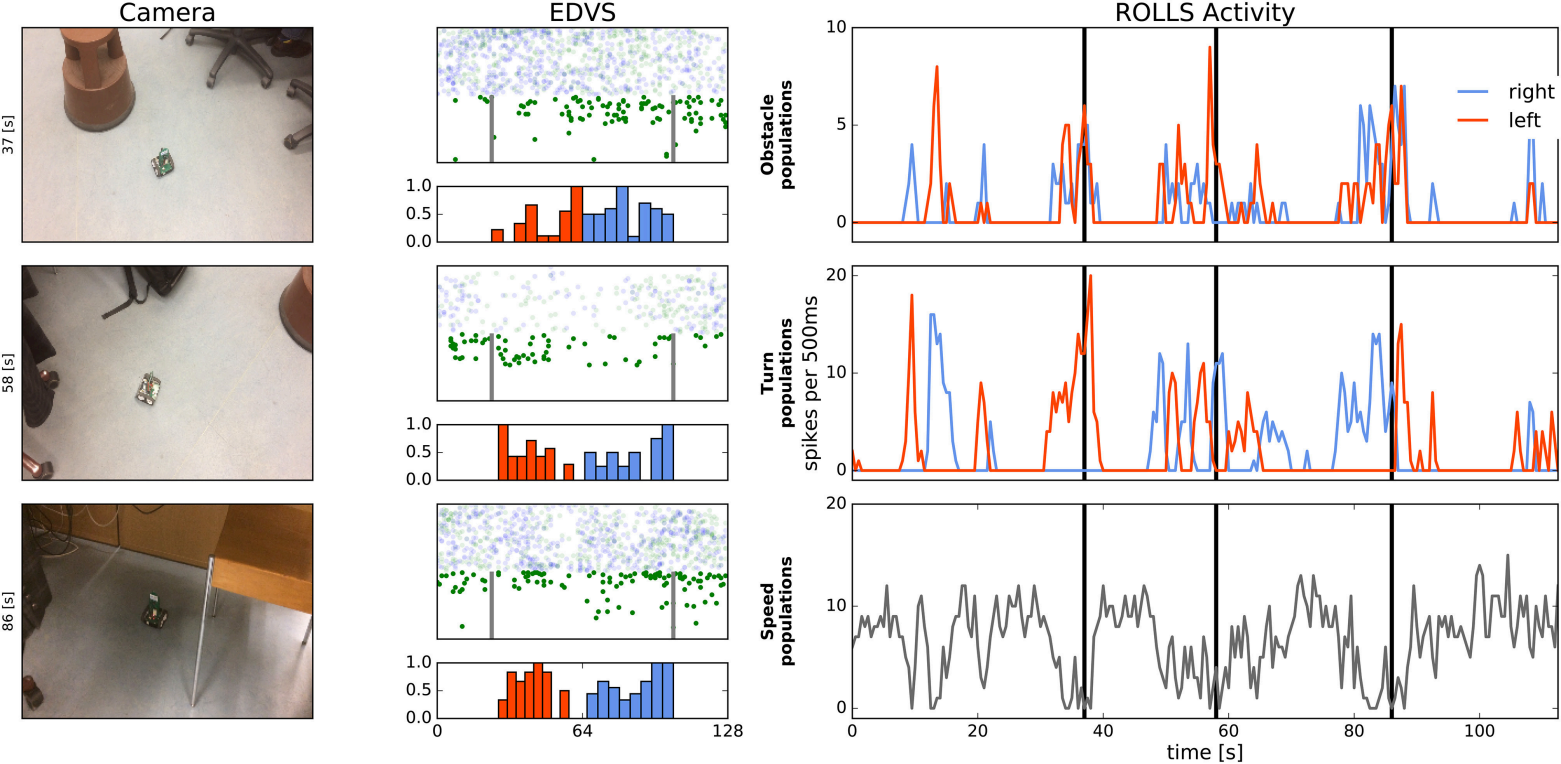
Robot driving in the office environment. **Left:** Snapshots from the video camera showing robot at three time points during the experiment. **Middle:** Events from the DVS camera and histogram of these events, binned over 500 *ms* in columns in the region between two vertical lines, which were used to drive obstacle populations on the ROLLS. Each pair of the eDVS events and histogram corresponds to the time point of the video frame in the **Left** column. Note that 80% of events are randomly dropped here and only “on” events are shown in the relevant region (lower part of the screen). Events above the midline of the image sensor are shown with transparency (these events were not used for obstacle avoidance). **Right:** Activity of the obstacle (left and right), drive (left and right), and speed neuronal populations over time (summed activity across each population). Vertical lines mark time point that correspond to the video frames in the **Left** column.

Figure 13 shows an example of the Pushbot robot driving in the office environment. On the left, three snapshots from the video camera recording the driving robot are shown (full videos can be see in the Supplementary Material). The snapshots show the robot navigating the office environment with its task being to avoid collisions. The middle column of plots shows pairs of eDVS events, accumulated over 500 *ms* around the moment in time in the corresponding snapshot on the left, and respective histograms of events from the center region, used for obstacle avoidance. Events above the mid-line of the eDVS field-of-view are shown with transparency to emphasize that they were not used for obstacle avoidance: only events from the region of the eDVS field-of-view between the two vertical lines in Figure 13 were used.

Histograms below the eDVS plots show the events from this region of the field of view, summed over the eDVS columns. These events drive the obstacle left (red colored part of the histogram) and obstacle right (blue part of the histogram) neuronal populations on the ROLLS chip.

The right column shows activity of the neuronal populations on the ROLLS chip over time, as in the previous figures. Black vertical lines mark time moments that correspond to the three snapshots in the left column. These plots allow to see that although the left and right obstacle populations are often activated concurrently, only one of the drive populations (either left or right) is active at any moment, leading to a clear decision to turn in either direction in the presence of perceived obstacles. The speed plot shows that movement of the robot is not very smooth—it slows down and accelerates often based on the sensed presence of obstacles. This behavior is improved in the modified architecture, briefly described in Section 3.4.

When driving around the office, robot faced very different lighting conditions, as can be seen already in the three snapshots presented here. This variation in lighting conditions did not effect obstacle avoidance in most cases, since the DVS is sensitive to relative change of each pixel's intensity, which varies less than the absolute intensity when the amount of ambient light changes. However, in an extreme case, shown in the lower snapshot in Figure 13, the robot collided with the metal foot of the chair. This was the only collision recorded.

### 3.7. Target acquisition

In addition to obstacle avoidance we also tested target acquisition in ten experiments using a second robot with a blinking LED as target. The robot successfully turns and drives toward the target every time (at speed and turn factors = 0.5). In 8 out of 10 experiments the target is recognized as an obstacle when approached and is avoided; in two experiments, the robot failed to recognize target as obstacle after approaching it.

Obviously, the simple visual preprocessing that we used did not allow us to distinguish the target from obstacles (other than through their position in the upper or lower part of the field-of-view of the DVS). Moreover, we would need an object detection algorithm to detect the target and segregate it from the background. This vision processing is outside the scope of our work, but there is a multitude of studies going in this direction (Moeys et al., 2016) using modern deep/convolutional neural networks learning techniques.

**Figure 14:**
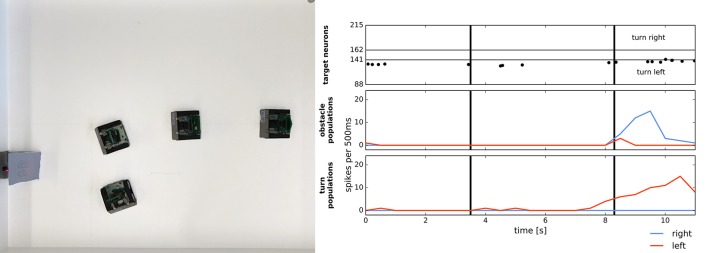
Simple target acquisition: single stationary target. **Left:** Overlay of video frames from the overhead camera. The robot approaches a stationary target on the left-hand side of the arena from right to left. The robot turns left toward the target until it perceives it as an obstacle and makes an obstacle avoidance maneuver. **Right:** Time-course of the spiking activity (raster plot) of the target-representing (WTA) neurons on the ROLLS chip (top plot) and summed (over 500 ms and over populations) activity of neurons in obstacle representing and drive populations on the ROLLS chip. Vertical lines mark time points that correspond to two middle positions of the navigating robot.

Figure 14 shows target acquisition for a static target and demonstrates that the robot can approach the target object. At a short distance, the obstacle component takes over and the robots turns away after approaching the target. The figure shows the overlayed snapshots from the overhead camera, showing how the robot turns toward the second robot, standing on the left side of the image. When getting close to the second robot (~10 cm), the robot perceives the target as an obstacle, which has a stronger contribution to its movement dynamics and the robot turns away. On the left, the spiking activity of the target representation on the ROLLS chip is shown (raster plot where each dot represents a spike^5^). We can see that the robot perceives its target consistently on the left. After the eighth second, the obstacle contribution on the right becomes dominant and the robot turns left strongly.

**Figure 15:**
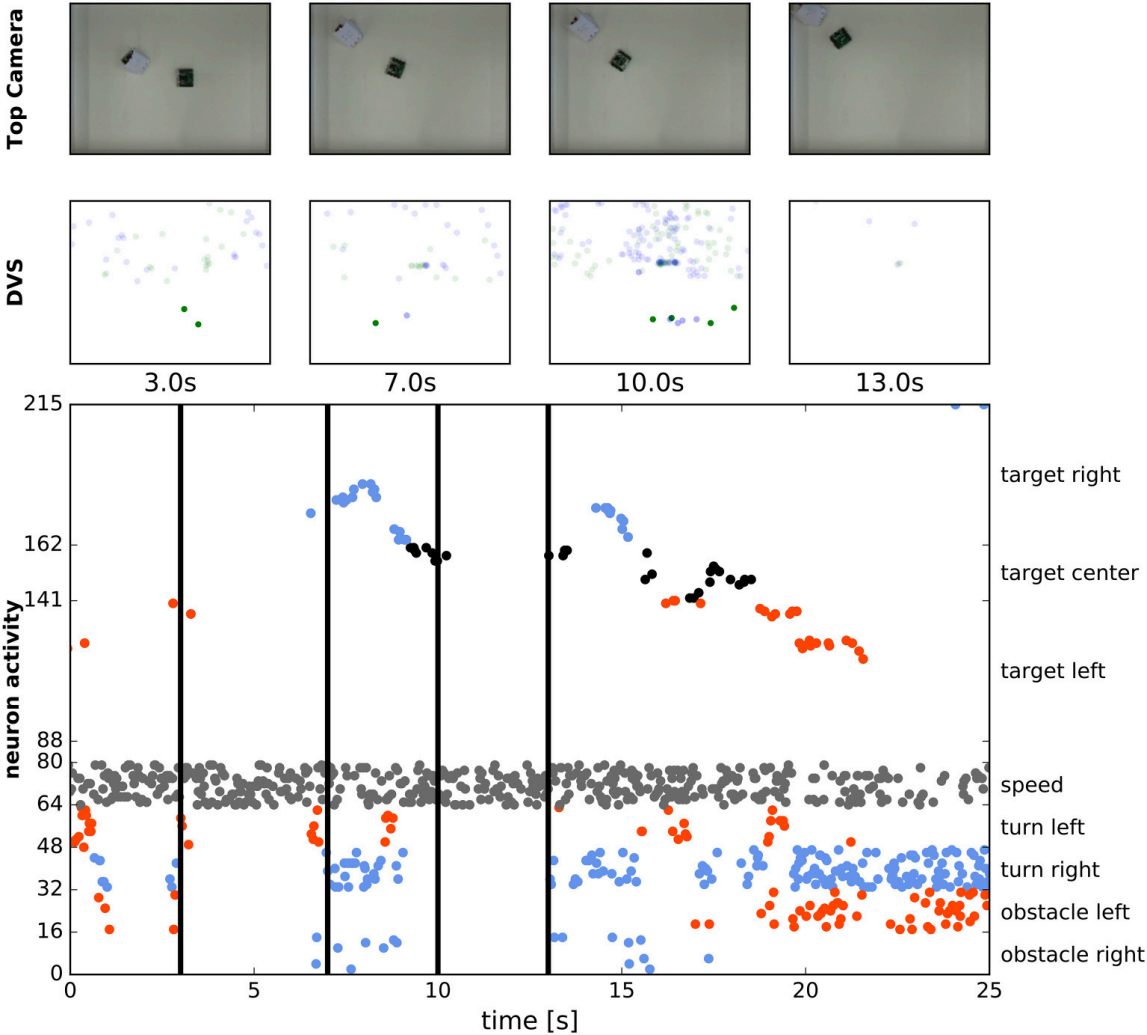
Chasing a moving target. **First row:** Snapshots from the overhead camera showing the robot controlled by the ROLLS chip chasing a manually controlled robot. **Second row:** Summed eDVS events from 500 ms time windows around the same moments. Events from the upper part were used for target acquisition, events from the lower part—for obstacle avoidance. **Bottom row:** Spiking activity of all neurons on the ROLLS chip over the time of the experiment. Vertical line show the moments in time, selected for the first two rows. Red dots are spikes from the “left” populations and blue dots are spikes from the “right” populations.

Figure 15 shows how the robot can chase a moving target. We have controlled the second Pushbot remotely and have turned its LED on (at 200 Hz, 75% on-time). The LED provided a rather strong (though spatially very small) input to the DVS of the second, autonomously navigating robot. This input was integrated by our target WTA (DNF) population, which, however, also received a large amount of input from the background (in the upper part of the field of view the robot could see behind the arena's walls). Input from the localized LED was stronger and more concise than more distributed input from the background and such localized input was enhanced by the DNF's (WTA's) lateral connections. Consequently, the respective location in the target WTA formed a “winner” (localized activity bump in the DNF terminology) and inhibited the interfering inputs from other locations.

In the figure, four snapshots of the video recording the two robots are shown (top row). The leading robot was covered with white paper to reduce interference from the obstacle avoidance dynamics as the robots get close to each other (the space in the arena and the small size of the blinking LED forced us to put the robots rather close to each other, so that the target robot could be occasionally perceived as an obstacle).

In the second row in Figure 15, the summed over 500 ms events of the DVS are shown, around the same time points as the snapshots. Only the upper part of the field-of-view was considered for target acquisition. This part is very noisy, since the robot “sees” outside the arena and perceives objects in the background, which made target acquisition very challenging. Still, the blinking LED provided the strongest input and in most cases the target DNF was able to select its input as the target and suppress the competing inputs from the background—see activity of neurons in the target DNF in the bottom plot.

This last plot shows spiking activity of 215 neurons of the ROLLS chip, used to drive the robot (we don't show the constantly firing *n*_*exc*_ population here). We can see that the target DNF (WTA) successfully selects the correct target in most cases, only loosing it from sight twice, as the robot receives particularly many DVS events from the background during turning. The lower part of this raster plot shows activity of the obstacle populations, the drive populations, and the speed population, thus the dynamics of the whole architecture can be seen here.

## 4. Discussion

This paper presents a neuronal architecture for reactive obstacle avoidance and target acquisition, implemented using a mixed-signal analog/digital neuromorphic processor (Qiao et al., 2015) and a silicon retina camera DVS as the only source of information about the environment. We have demonstrated that the robot, controlled by interconnected populations of artificial spiking neurons, is capable of avoiding multiple objects (including moving objects) at an average movement speed (up to 0.35 m/s with our proof of concept setup). We have also demonstrated that the system works in a real-world office environment, where background clutter poses a challenge for the DVS on a moving vehicle, creating many distracting events. We demonstrated that also the target acquisition neural architecture can cope well with this challenge, which was relevant even in the robotic arena. The distributed DNF representation of the target, supported by lateral interactions of the WTA neuronal population, enabled robust detection and reliable selection of the target against background.

The reactive approach to obstacle avoidance that we adopt in this work has a long history of success, starting with the neurally inspired turtle robot more than half a century ago, as reviewed by Holland (1997). Later, Valentino Braitenberg analyzed a number of hypothetical vehicles, or creatures, that use reactive control to produce complex behaviors (Braitenberg, 1986). His controllers were realized as simple “nervous systems” that directly linked the sensors to the motors of the vehicle. Using similar sensorimotor, or behavioral modules as building blocks, Rodney Brooks developed a behavior-based controller paradigm for roaring vehicles, known as “subsumption architecture” (Brooks, 1991). Although this framework did not scale well for complex tasks and is not ideally suited for online learning methods, this type of controller is at the heart of highly successful real-world robotic systems such as the autonomous vacuum cleaners, and has been adopted, to some extent, in a wide range of impressive controllers for autonomous robots (e.g., Khansari-Zadeh and Billard, 2012).

The dynamical systems approach to robot navigation (Schöner et al., 1995) is an attempt to mathematically formalize reactive control for autonomous robots using differential equations that specify attractors and repellors for behavioral variables that control the robot's heading direction and speed (Bicho et al., 2000). In this framework, obstacle avoidance has been integrated with target acquisition and successful navigation in an unknown environment has been demonstrated both for vehicles and robotic arms (Reimann et al., 2011). This approach is similar to another successful reactive approach to obstacle avoidance: the potential field approach (e.g., Haddad et al., 1998), in which the target creates a global minimum in a potential that drives the robot, whereas obstacles create elevations in this potential. However, the use of Cartesian space instead of robot-centered velocity space used in this potential field approach makes it prone to getting trapped in local minima.

In mixed-signal analog /digital neuromorphic hardware, the neuronal dynamics is taken care of by the physics of analog electronic circuits, avoiding loosing digital computational resources on simulating them. Thus, neuromorphic implementation of simple biologically inspired obstacle-avoidance architectures can lead to low-latency (on the order of microseconds) and power-efficient (on the order of milliwatts) solutions, analogous to the ones used by insects. In contrast, more conventional obstacle-avoidance systems require a substantial amount of computing resources to process and store sensory data, detect obstacles, and compute motor commands. Neuromorphic implementation of such low-level processing will allow to use analog sensory signals directly, avoiding their digital representation and storage, while at the same time allowing to build complex neural-network based computing architectures, that could be used for solving cognitive tasks, such as task planning, map building, or object recognition.

We consider the work proposed as a first feasibility study, which still has a number of limitations that we will address in our future work. The main limitation is variability of neuronal behavior because of parameter drift on the analog hardware: the parameters of the hardware neural network change the network properties as the experimental setup conditions (temperature, humidity, etc.) change. This is a serious limitation of the hardware used, which makes in challenging to implement complex architectures that have to balance contributions of different behavioral modules (e.g., controlling turning and forward velocities, or obstacle avoidance and target acquisition). We are currently working on algorithms and methods for automatically re-tuning these parameters in a principled fashion with optimization and machine learning techniques. In addition, we are designing new versions of the neuromorphic hardware with on-board stabilization of the chip parameters, and more resources for simplifying the fine-tuning process of the architectures. However, approach employed here—use of populations of artificial neurons in place of single nodes in the architecture—allowed us to generate behavior with the state of the art analog neuromorphic hardware.

Apart from the hardware limitations, our simple architecture currently allows robust obstacle avoidance at moderate speeds (~0.35 m/s). Since the robot slows down when an obstacle is detected, movement appears to be “jerky.” Although the smoothness of the robot movement could be improved by tuning the coupling strength between the obstacle and drive populations, the best solution would involve improving the visual pre-processing stages. In our setup, the DVS detects local contrast changes and produces different amount of events depending on the objects in the environment, but also modulated by the robot translational and rotational movements. Currently we ignore about 80% of all DVS events to remove both noise and to reduce bandwidth. This very basic strategy improves the signal to noise ratio, because the architecture enhances the spatially and temporally coherent inputs and suppresses the effect of random inputs. However, we plan to study a more principled approach to pre-processing and noise reduction, and to investigate other biologically inspired architectures for obstacle avoidance, for example inspired by the fly's EMD (Elementary Motion Detector) (Hassenstein and Reichardt, 1956) or the locust's LGMD (looming detector Lobula Giant Movement Detector) (Gabbiani et al., 2002; Rind and Santer, 2004). We are currently working on neuromorphic implementation of these algorithms (Milde et al., 2016; Salt et al., 2017).

Moreover, the 500 ms time window that we used to create plots of DVS events and average spiking activity was also used in our controller for counting spikes when calculating motor commands, sent to the robot. In our preliminary experiments on optimizing the controller, we have reduced this time window to 50 ms and, more importantly, replaced it with a sliding-window calculation of the average firing rate of the drive and speed neuronal populations. A more principled solution to this problem would be development of a more direct hardware interface between the spiking neuromorphic processor and the robot's motors, so that spikes can control the motor rotation directly, as suggested by Perez-Peña et al. (2013).

Our target acquisition network can also be further improved: the main strategy will be to introduce target representations in a reference frame that moves with the robot, but has a fixed orientation. Such representation will allow the robot to turn back to a target that has been lost from sight due to an obstacle avoidance maneuver. Furthermore, increasing the strength of lateral interactions in the WTA (DNF) population will allow to stabilize the target representation, allowing it to form a “working memory,” which will support target acquisition behavior in cluttered environments. To still make the system reactive and allow it to follow the visible target, control of the strength of lateral interactions will be introduced, increasing their strength when target is being lost from view and decreasing their strength when the target is visible. Detecting the target based on its features perceived with a DVS is a separate topic of ongoing research both in our lab and worldwide (e.g., Lagorce et al., 2015).

Despite of this list of necessary improvements, our neuromorphic architecture is an important stepping stone toward robotic controllers, realized directly in neurally inspired hardware, being the first architecture for closed-loop robot navigation that uses analog neuromorphic processor and minimal preprocessing of visual input, obtained with a silicon retina DVS. Such neuromorphic controllers may become an energy efficient, fast, and adaptive alternative to conventional digital computers and microcontrollers used today to control both low-level and cognitive behaviors of robots. While *neural network* implementations using the conventional computing architecture are typically time- and energy consuming, implementation of neuronal architecture using analog neuromorphic hardware approaches the efficiency of biological neural networks. Building neuronal models for higher cognitive function using, for instance, the framework of Dynamic Neural Fields (Sandamirskaya, 2013) or the Neuro-Engineering Framework (Eliasmith, 2005), will allow to add more complex behaviors to the robot's repertoire, e.g., finding a particular object, grasping and transporting it, as well as map formation and goal-directed navigation, which is the goal of our current research efforts.

## Author contributions

MM: conceptualization of the model, analysis of the results, writing up. HB and AD: implementation of combined obstacle avoidance and target acquisition, experiments, results analysis, writing up; DS: implementation of first version of obstacle avoidance, parameter tuning on the chip, state of the art analysis; JC: support with robotic hardware and middleware, analysis of the results, writing up; GI: support with neuromorphic hardware, and state of the art and result analysis, writing up; YS: conceptualization of the model, development of the architecture, experiment design, analysis of the results, embedding in the literature, discussion of the results, writing, and overall supervision of the project.

### Conflict of interest statement

The authors declare that the research was conducted in the absence of any commercial or financial relationships that could be construed as a potential conflict of interest.

## Footnotes

^1^https://www.parallella.org

^2^http://inilabs.com/products/pushbot

^3^We dropped 80% of DVS events randomly in our architecture; moreover, we only used 5% of all remaining events for plotting.

^4^Only 5% of the ROLLS spikes (every 20th spike) are shown in all our plots.

^5^Remember, that only 5% (every 20th) of all spikes from the ROLLS processor are shown.
