# Supplementary material for: Obstacle Avoidance and Target Acquisition for Robot Navigation Using a Mixed Signal Analog/Digital Neuromorphic Processing System
Source: Front Neurorobot. 2017 Jul 11;11:28. doi: 10.3389/fnbot.2017.00028 (PMC5507184; doi:10.3389/fnbot.2017.00028)
Supplement: Supplementary file 14 [file Appendix.pdf]

SUPPLEMENTARY MATERIAL

Appendix A: Neuronal equations

The dynamics of the spiking neurons on the ROLLS chip can be approximated by the differential equation Eqs. (1-3), obtained by performing circuit analysis:

$$\tau \frac{dImem}{dt} = \frac{\frac{I_{th}}{I_\tau}(I_{in} - I_{ahp} - I_\tau) + \frac{I_a}{I_\tau}(I_{mem} + I_{th}) - I_{mem}(1 + \frac{I_{ahp}}{I_\tau})}{(1 + \frac{I_{th}}{I_{mem}})}, \tag{1}$$

$$\tau_{ahp} \frac{dI_{ahp}}{dt} = \frac{I_{thahp}}{I_{\tau ahp}} I_{Ca} u(t) - I_{ahp}, \tag{2}$$

where I_{mem} is the membrane potential, I_{ahp} is the adaptation current, $u(t)$ is a step function that is one during spikes and zero otherwise, I_τ and $I_{\tau ahp}$ are time constant currents, I_th and I_{thahp} are currents through N-type MOSFETs, τ and τ_{ahp} are time constants, and I_{in} is the input current from the synapses.

The time constants are dependent on the time constant currents and can be calculated by:

$$\tau = \frac{C_{mem} U_T}{\kappa I_{tau}}, \tag{3}$$

where κ is a MOSFET property, U_T is the thermal potential, and C_{mem} is the membrane capacitance. τ_{ahp} is calculated similarly except it substitutes I_τ with $I_{\tau ahp}$ and C_{mem} with C_p.

These equations approximate an adaptive integrate-and-fire dynamics (Brette and Gerstner, 2005) .

Appendix B: Biases of the ROLLS chip used in our experiments

Table 1 shows the biases used for our experiments to set-up non-plastic connections between the neuronal populations; Table 2 shows biases for the integrate-and-fire neurons on chip. Each bias corresponds to a current, supplied to the neuronal circuits and is calculated as Range $\times$ Value, where letters near the range mean the order of magnitude: "p" – piko, "n" – nano, "u" – micro (see (Qiao et al., 2015) for details of the circuit and functional meaning of the biases). The biases are set using software and FPGA-mapping, implemented on the Parallella board.

Table 1. Hardware biases for the non-plastic synapses

Bias name	Range (A)	Value	Flags
NPA_PWLK_P	820p	200	H
NPA_WEIGHT_STD_N	15p	15	H N
NPA_WEIGHT_EXC_P	820p	123	H
NPA_WEIGHT_EXC0_P	0.4u	15	H
NPA_WEIGHT_EXC1_P	0.4u	82	H
NPDPIE_THR_P	820p	38	H
NPDPIE_TAU_P	105p	22	H
NPA_WEIGHT_INH_N	820p	82	H N
NPA_WEIGHT_INH_N0	820p	200	H N
NPA_WEIGHT_INH_N1	6.5n	71	H N
NPDPII_TAU_P	15p	51	H
NPDPII_THR_P	820p	177	H

Table 2. Hardware biases for integrate-and-fire neurons

Bias name	Range (A)	Value	Flags
IF_RST_N	15p	17	H N
IF_BUR_P	50p	56	H N
IF_ATHR_N	15p	0	H N
IF_RFR1_N	820p	50	H N
IF_RFR2_N	820p	50	H N
IF_AHW_P	15p	0	H
IF_AHTAU_N	820p	37	N
IF_DC_P	15p	0	H
IF_TAU2_N	105p	77	N
IF_TAU1_N	105p	100	N
IF_NMDA_N	15p	17	H N
IF_CASC_N	15p	17	H N
IF_THR_N	820p	100	H N

The eight different values of the synaptic weights that we used in our architecture (-4 : 4) are obtained combining the NPA_WEIGHT_INH_N, NPA_WEIGHT_INH_N1, and NPA_WEIGHT_INH_N2 biases for negative weights and the NPA_WEIGHT_EXC_P, NPA_WEIGHT_EXC_P1, and NPA_WEIGHT_EXC_P2 biases for positive weights:

$$
\begin{aligned}
&1 = NPA\_WEIGHT\_EXC\_P \\
&2 = NPA\_WEIGHT\_EXC\_P + NPA\_WEIGHT\_EXC\_P1 \\
&3 = NPA\_WEIGHT\_EXC\_P + NPA\_WEIGHT\_EXC\_P2 \\
&4 = NPA\_WEIGHT\_EXC\_P + NPA\_WEIGHT\_EXC\_P1 + NPA\_WEIGHT\_EXC\_P2 \\
&-1 = NPA\_WEIGHT\_INH\_N \\
&-2 = NPA\_WEIGHT\_INH\_N + NPA\_WEIGHT\_INH\_N1 \\
&-3 = NPA\_WEIGHT\_INH\_N + NPA\_WEIGHT\_INH\_N2 \\
&-4 = NPA\_WEIGHT\_INH\_N + NPA\_WEIGHT\_INH\_N1 + NPA\_WEIGHT\_INH\_N2
\end{aligned}
$$

REFERENCES

Brette, R. and Gerstner, W. (2005). Adaptive exponential integrate-and-fire model as an effective description of neuronal activity. *J. Neurophysiol.* 94, 3637–3642. doi:10.1152/jn.00686.2005

Qiao, N., Mostafa, H., Corradi, F., Osswald, M., Sumislawska, D., Indiveri, G., et al. (2015). A Reconfigurable On-line Learning Spiking Neuromorphic Processor comprising 256 neurons and 128K synapses. *Frontiers in neuroscience* 9
